# Cosmetic satisfaction and patient-reported outcomes following surgical treatment of single-suture craniosynostosis: a systematic review

**DOI:** 10.1007/s00381-023-06063-3

**Published:** 2023-07-21

**Authors:** Vita M. Klieverik, Ash Singhal, Peter A. Woerdeman

**Affiliations:** 1 Department of Neurology and Neurosurgery, University Medical Center Utrecht, Heidelberglaan 100, 3584 CX Utrecht, The Netherlands; 2 Division of Pediatric Neurosurgery, British Columbia Children’s Hospital, Vancouver, BC Canada

**Keywords:** Craniosynostosis, Cosmesis, Cosmetic satisfaction, Patient-reported outcomes

## Abstract

**Purpose:**

This study provides a systematic review on cosmetic satisfaction and other patient-reported outcomes (PROMs) of patients who underwent surgical treatment of SSC.

**Methods:**

A systematic review of all articles published from inception to 1 June 2022 was performed. Articles were included if they reported on subjective assessment of cosmetic satisfaction or other PROMs by patients or their families using questionnaires or interviews.

**Results:**

Twelve articles, describing 724 surgical treatments of SSC, met the inclusion criteria. Cosmetic satisfaction was evaluated in the following ways: 1) use of the VAS score, binary questions or a 5-point scale to rate general, facial or skull appearance; 2) use of an aesthetic outcome staging in which personal opinion was added to the treating surgeon’s opinion; and 3) use of an evaluation of anatomical proportions of the skull and face. A trend towards an overall improvement in cosmetic satisfaction following surgical treatment of SSC was observed. Reported PROMs included general health, socioeconomic status, patients’ and their families’ rating of the normalcy and noticeability of their appearance and how much this bothered them, and patients’ answers to the Youth Quality of Life with Facial Differences (YQOL-FD) questionnaire. No clear overall trend of the reported PROMs was identified.

**Conclusion:**

This systematic review illuminates that there is a wide variation in outcomes for evaluating cosmetic satisfaction and other PROMs of patients who underwent surgical treatment of SSC, suggesting that further research is needed to develop an inclusive and uniform approach to assess these outcomes.

## Introduction

Craniosynostosis is a condition characterized by premature fusion of one or more cranial sutures [1–3]. The incidence of craniosynostosis is around 1 in 2500 live births [4]. Approximately 8% of patients suffer from a syndromic or familial form of craniosynostosis, but it mostly occurs as an isolated defect of one cranial suture, known as single-suture craniosynostosis (SSC) [2, 4]. SSC is often surgically treated to correct and prevent severe cosmetic deformity and to prevent potential impairments in brain and cognitive development [5]. Postoperative follow-up usually does not extent past school age because late sequelae of neuropsychological and cognitive impairments are usually not expected after surgical treatment [6, 7]. Nevertheless, patients with SSC may suffer from psychosocial difficulties, either during childhood or later on in life [7]. For example, a retained cosmetic deformity in these patients may draw negative attention from strangers and lead to rejection from peers [7]. Consecutively, this might negatively influence these patients’ level of self-esteem, sense of belonging, social behavior and experience, and overall health-related quality of life [7]. Therefore, evaluation of patients’ subjective assessment of their skull and facial appearance is important [8]. Furthermore, patient-reported outcomes (PROMs) such as self-esteem, social behavior and quality of life are other valuable outcome measures to evaluate. An inclusive and systematic review of recent literature on these outcomes is currently lacking. An overview of which outcomes concerning cosmetic satisfaction and other PROMs following surgical treatment of SSC are currently being reported may help guide clinical practice in deciding how to best evaluate these patients. Furthermore, these results provide insight into how surgical treatment of SSC affects patients’ lives, which may help optimize preoperative counseling and psychosocial care for these patients and their families. Therefore, the objective of the present study is to provide a systematic review of the literature on cosmetic satisfaction and other PROMs of patients who underwent surgical treatment of SSC.

## Methods

This systematic review was performed according to the Preferred Reporting Items for Systematic Reviews and Meta-Analyses (PRISMA) guidelines, an evidence-based set of criteria for reporting in systematic reviews and meta-analyses [9].

### Literature search

An extensive search in the electronic medical databases of PubMed and Embase has been performed to identify all relevant literature on cosmetic satisfaction and other PROMs of patients who underwent surgical treatment of SSC up to 1 June 2022. The full search term was as follows: (craniosynostosis) AND ((cosmesis) OR (cosmetic outcome) OR (aesthetic outcome) OR (patient-reported outcome) OR (subjective outcome)) [10].

### Study selection

Study selection was done by 2 independent authors (V.K., P.W.) and disagreements were solved by discussion including a third author (A.S.). First, the title and abstracts of all retrieved papers were screened. Titles or abstracts of studies that did not report on any postoperative outcomes of surgical treatment of SSC were excluded. Subsequently, the full texts of the remaining papers were thoroughly reviewed. Finally, the references of the studies yielded by the search and the “similar articles” feature of the electronic medical databases were reviewed to identify additional relevant studies.

Studies were included if they met the following criteria:

1. They reported on cosmetic satisfaction or other PROMs of patients who underwent surgical treatment of SSC.
2. These outcomes included subjective assessment of skull and facial appearance, self-esteem, social behavior or quality of life.
3. These outcomes were assessed by patients or their parents or caregivers themselves using questionnaires or interviews.
4. They were designed as prospective or retrospective cohort studies, case–control studies or clinical trials.

Studies were excluded if they reported on:

1. Anthropometric measurements or computed tomography (CT)-based morphometric parameters only.
2. Cosmetic outcomes assessed by independent raters, for example a panel of surgeons or laymen. This also included using the Whitaker classification for describing cosmetic outcomes, since this is often assessed by the treating surgeon instead of the patients themselves [11].
3. Outcomes of novel or experimental surgical treatments for SSC that are not routinely used in clinical practice.

### Data extraction

Data obtained from each study included 1) year of publication; 2) Center for Evidence-Based Medicine (CEBM) level of evidence; 3) number of patients with SSC; 4) type of SSC (sagittal, metopic or coronal); 5) type of surgical treatment for SSC; 6) mean age at surgical treatment; 7) mean duration of follow-up after surgical treatment; 8) method of outcome assessment; 9) by whom the outcome assessment was done; 10) time of outcome assessment; 11) types of outcome measures to study cosmetic satisfaction or other PROMs; and 12) results of these outcome measures. Data extraction was done by two independent authors (V.K., P.W.).

## Results

**Fig. 1:**
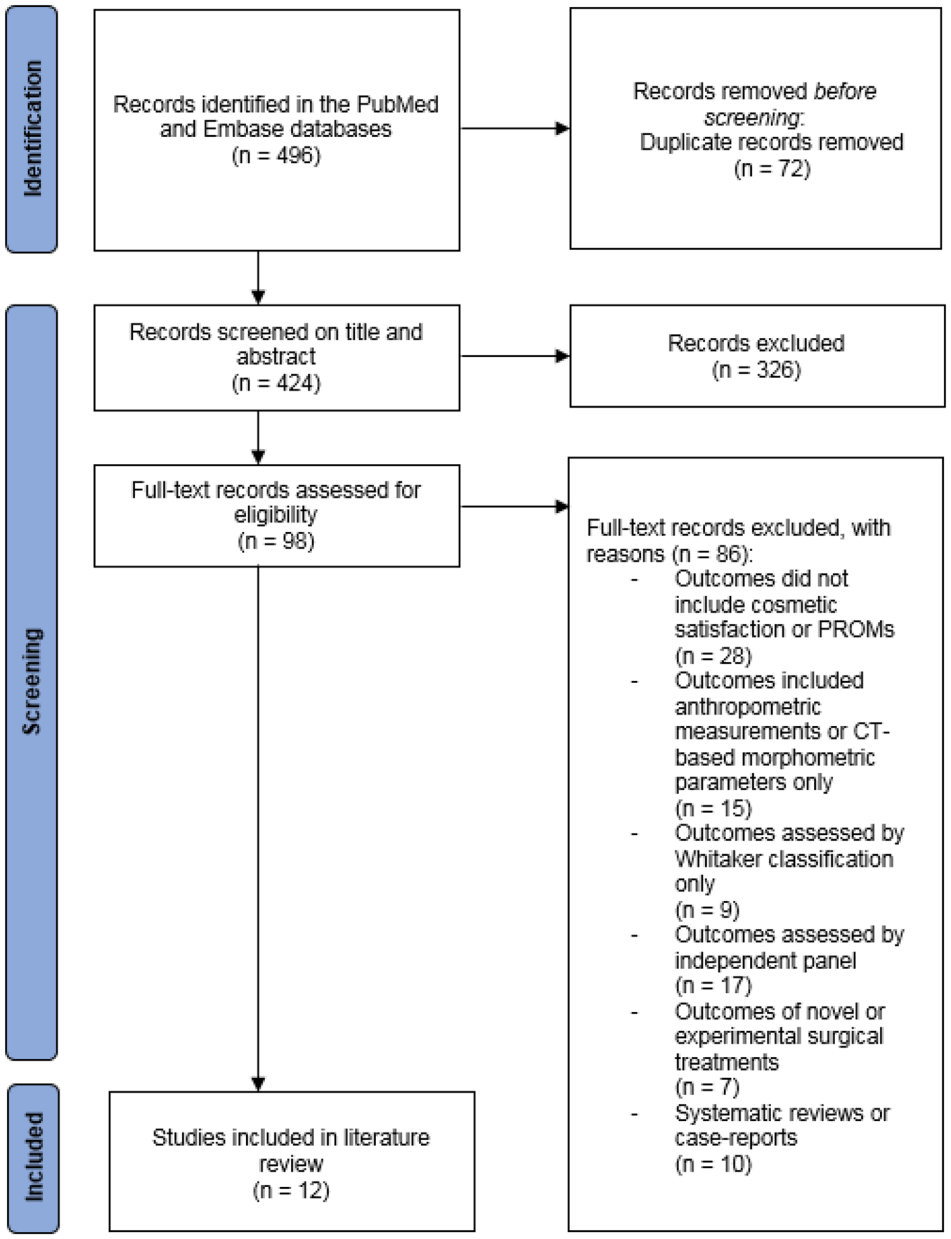
Flowchart depicting study selection

The search retrieved a total of 496 papers published from August 1982 to June 2022. Of these papers, 72 duplicates were removed. Next, 326 papers were excluded after reading the title and abstract alone and another 86 were excluded after reviewing the full text (Fig. 1). A total of 12 studies describing cosmetic satisfaction and other PROMs of patients who underwent surgical treatment of SSC met the inclusion criteria and were included in the literature review [7, 12–22].

Six of the 12 included studies met the CEBM criteria for level 2B as retrospective cohort studies [13, 15, 16, 18–20], 3 met criteria for level 3B as case–control studies [7, 21, 22] and the remaining studies met criteria for level 4 as case series [12, 14, 17]. All studies collectively described 724 patients who underwent surgical treatment of SSC (Table 1). Ten of the 12 included studies reported on the type of surgical treatment performed, which included open reconstructive surgery (ORS), endoscopy-assisted suturectomy, extended midline strip craniectomy (EMSC), modified pi procedure (mPP), frontal orbital advancement (FOA), cranial vault remodelling (CVR), strip craniectomy (ST), suturectomy (ST), orbitofrontal bandeau resection (OFBR), spring-assisted cranioplasty (SAS), pi procedure (PP) and burring of the metopic ridge (MRB) [7, 12, 14–20, 22]. The age at treatment ranged from 0.3 months to 12 years [7, 12, 14, 18, 19, 22] and the weighted mean was 6.4 months [7, 12, 14–17, 19, 20]. The duration of follow-up ranged from 6 months to 37 years [7, 12, 17–20] and had a weighted mean of 7.9 years [7, 12, 17, 19, 20]. Table 2 presents a summary of reported outcome measures of cosmetic satisfaction and other PROMs of patients who underwent surgical treatment of SSC. Nine papers presented results on cosmetic satisfaction [7, 12, 15, 17–22] and 4 papers reported other PROMs [7, 13, 14, 16]. The time of outcome assessment ranged from 3 months postoperatively to a minimum age of 18 years postoperatively [7, 12–14, 16–19, 22].

**Table 1:** Included studies of cosmetic satisfaction and other PROMs of patients who underwent surgical treatment of SSC

Author & Year	CEBM Level of Evidence	Number of patients	Type of SSC	Type of treatment	Mean age at treatment in months (range)	Mean follow-up in months (range)
Svalina et al. (2022) [21]	3B	41 8	Sagittal Metopic	*N/A*	*N/A*	*N/A*
Dalton et al. (2022) [13]	2B	248	Sagittal Metopic Coronal	*N/A*	*N/A*	*N/A*
Baş and Baş (2021) [12]	4	18	Metopic	ORS EAS	6.1 (1.0 – 9.0)	18.0 (12.0 – 37.0)
Millesi et al. (2021) [19]	2B	99	Sagittal	EMSC mPP	5.5 (1.6 – 20.0)	43.2 (12.0 – 156.0)
Kampf et al. (2020) [17]	4	20	Metopic	FOA	7.9 (*N/A*)	48.6 (12.0 – 110.0)
Gabrick et al. (2020) [14]	4	21	Coronal	CVR and FOA	7.8 (4.7 – 17.1)	*N/A*
Salokorpi et al. (2019) [7]	3B	40	Sagittal	SR ST H-plasty	5.7 (0.3 – 45.0)	318.0 (204.0 – 444.0)
Joly et al. (2016) [16]	2B	9 8	Metopic Coronal	ST and OFBR	5.9 (*N/A*) 8.1 (*N/A*)	*N/A*
Mutchnick and Maugans (2012) [20]	2B	18	Sagittal	CVR	2.3 (*N/A*)	16.4 (6.0 – 38.0)
Windh et al. (2008) [22]	3B	40	Sagittal	SAS PP	3.5 (2.5 – 5.5) 7.1 (4.0 – 15.5)	*N/A*
Kelleher et al. (2007) [18]	2B	44	Metopic	FOA MRB	12.0 (4.0 – 144.0)*	60.0 (12.0 – 192.0)*
Guimarães et al. (2001) [15]	2B	110	Sagittal	mPP	7.7 (*N/A*)	*N/A*
Total		724			6.4 (0.3 – 144.0)	95.3 (6.0 – 444.0)

*PROMs* patient-reported outcomes, *SSC* single-suture craniosynostosis, *CEBM* Center of Evidence-Based Medicine, *ORS* open reconstructive surgery, *EAS* endoscopy-assisted suturectomy, *EMSC* extended midline strip craniectomy, *mPP* modified pi procedure, *FOA* frontal orbital advancement, *CVR* cranial vault remodelling, *SR* strip craniectomy, *ST* suturectomy, *OFBR* orbitofrontal bandeau resection, *SAS* spring-assisted cranioplasty, *PP* pi procedure, *MRB* metopic ridge burring, *N/A* not available

*Median

**Table 2:** Summary of reported outcome measures of cosmetic satisfaction and other PROMs of patients who underwent surgical treatment of SSC

Author & Year	Outcome assessment method	Outcome assessment by	Time of outcome assessment	Type of outcome measures	Results of outcome measures
Svalina et al. (2022) [21]	Questionnaire	Patients	*N/A*	Using VAS score: **1.** Satisfaction with appearance in general **2.** Satisfaction with facial aesthetics	Correlation coefficients (p-value) between facial asymmetry parameters and VAS scores: **1.** -0.23 (0.016) for forehead AD and VAS score for Q1, 0.23 (0.016) for forehead SP and VAS score for Q1 **2.** -0.20 (0.03) for forehead AD and VAS score for Q2, 0.23 (0.013) for forehead SP and VAS score for Q2
Dalton et al. (2022) [13]	Questionnaire	Parents and patients	Parents: prospective data at 3 months postoperatively, retrospective data after many years postoperatively Patients: at a minimum age of 7 years	Using 10-point scale: *Parents:* **1.** “How noticeable is your child’s head shape to other people?” **2.** “How much does your child’s head shape bother you?” **3.** Rating of whether surgery made a difference to their child’s head shape *Patients:* **4.** “How noticeable is your head shape to other people?” **5.** “How much does it bother you?” *Both:* **6.** Comparison of parents’ and patients’ rating of “How noticeable is your (child’s) head shape to other people?” **7.** Comparison of parents’ and patients’ rating of “How much does it bother you?”	Mean decrease in points pre- versus postoperatively (p-value): **1.** 3.7 points for prospective data, 5.7 points for retrospective data (< 0.001) **2.** 4.3 points for prospective data, 4.9 points for retrospective data (< 0.001) Mean points: **3.** 9.5 points Mean difference in points between younger (7 – 11 years) and older (> 11 years) children (p-value): **4.** 1.22 points (0.001) **5.** 1.12 points (0.002) Results for comparisons (p-value): **6.** Patients report more noticeability than parents (0.013 for younger children, 0.02 for older children) **7.** Patients are more bothered by head shape than parents (0.72 for younger children, 0.02 for older children)
Baş and Baş (2021) [12]	Interview	Parents	At a minimum of 12 months postoperatively	Using aesthetic outcome staging: **1.** Stage I = excellent **2.** Stage II = good **3.** Stage III **4.** Stage IV	Proportions of patients in different stages: **1.** Stage I = 94.4% **2.** Stage II = 5.6% **3.** Stage III = 0% **4.** Stage IV = 0%
Millesi et al. (2021) [19]	Interview	Parents	At 12 months postoperatively	Using classification: **1.** Perfect postoperative result with anatomically normal proportions **2.** Anatomically normal proportions with minor outline irregularities **3.** Light deficit in either height or width **4.** Distinct deficit in height and/or width **5.** Still dolichocephalic	Proportions of patients in different classes: **1.** 66% **2.** 23% **3.** 5% **4.** 3% **5.** 3%
Kampf et al. (2020) [17]	Interview	Parents	At a minimum of 12 months postoperatively	Using aesthetic outcome staging: **1.** Stage I = excellent **2.** Stage II = good **3.** Stage III **4.** Stage IV	Proportions of patients in different stages: **1.** Stage I = 80% **2.** Stage II = 20% **3.** Stage III = 0% **4.** Stage IV = 0%
Gabrick et al. (2020) [14]	Questionnaire	Patients	At a minimum age of 9 years	Using YQOL-FD domains: **1.** Positive consequences **2.** Coping **3.** Negative consequences **4.** Negative self-image **5.** Stigma	Mean ± SD score of patients in different YQOL-FD domains: **1.** 38.4 ± 27.8 points **2.** 26.6 ± 33.9 points **3.** 16.1 ± 21.6 points **4.** 8.1 ± 14.1 points **5.** 8.5 ± 20.4 points
Salokorpi et al. (2019) [7]	Questionnaire	Patients	At a minimum age of 18 years	Using VAS score: **1.** “How satisfied are you with your current facial appearance?” Using self-reported questionnaire: **1.** “Is there something that bothers you in your facial appearance?” **2.** Rating of whether the patients’ scar bothered them **3.** Rating of general health with attention to medical history of headaches **4.** Rating of presence of mental disturbances **5.** Rating of educational attainment **6.** Rating of employment status **7.** Rating of relationship or marital status **8.** Rating of housing status	Mean VAS score (range): **1.** 75 mm (29 – 100 mm) Proportions of patients who answered “yes”: **1.** 32.5% **2.** 10.0% Proportions of other ratings: **3.** 22.5% suffered from migraine, 57.5% suffered from other occasional headaches **4.** 27.5% suffered from mental disturbances **5.** 22.5% did not obtain professional education **6.** 12.5% were unemployed **7.** 52.5% were in a permanent relationship **8.** 42.5% lived in own real estate
Joly et al. (2016) [16]	Interview	Parents and patients	At a mean age of 14 years	Using binary responses to: *Parents:* **1.** Rating of whether they felt that their child’s skull was normal *Patients:* **2.** “Do you find your skull normal?” **3.** “Do other children make comments about it at school?”	Proportions of parents or patients who answered “yes”: **1.** 35.3% **2.** 64.7% **3.** 35.3%
Mutchnick and Maugans (2012) [20]	Interview	Parents	*N/A*	Using binary responses to: **1.** “Are you pleased with the cosmetic outcome?” **2.** “Are you happy to have avoided a molding helmet?” **3.** “Have you wondered if a helmet would have made the head shape better?” **4.** “Are you worried that repeat surgery will be needed?”	Proportions of parents who answered “yes”: **1.** 86% **2.** 93% **3.** 71% **4.** 86%
Windh et al. (2008) [22]	Questionnaire	Parents	At a minimum age of 3 years	Using 5-point scale: **1.** Rating of appearance of skull length **2.** Rating of appearance of skull width **3.** Rating of appearance of forehead **4.** Rating of appearance of neck Using VAS score: **1.** Rating of satisfaction of skull shape **2.** Rating of whether they would recommend the procedure **3.** Rating of whether they would accept the procedure again if needed	Mean ± SD points: **1.** 4.3 ± 1.0 points **2.** 4.2 ± 1.1 points **3.** 4.4 ± 0.9 points **4.** 4.4 ± 0.8 points Mean ± SD VAS score: **1.** 5.6 ± 7.5 mm **2.** 1.9 ± 0.6 mm **3.** 12.4 ± 4.3 mm
Kelleher et al. (2007) [18]	Questionnaire	Parents and patients	At a minimum of 12 months postoperatively	Using 5-point scale: **1.** Rating of satisfaction with head shape **2.** Rating of satisfaction with bicoronal scar	Proportions of patients in different ratings: **1.** Excellent in 29%, very good in 40%, good in 31%, fair in 0%, poor in 0% **2.** Excellent in 48%, very good in 28%, good in 24%, fair in 0%, poor in 0%
Guimarães et al. (2001) [15]	Questionnaire	Parents	*N/A*	Using 5-point scale: **1.** Rating of appearance of skull length **2.** Rating of appearance of skull width **3.** Rating of shape of forehead **4.** Rating of shape of occiput Using VAS score: **1.** Rating of general aesthetic appearance of the skull	Mean ± SD points: **1.** 4.1 ± 1.0 points **2.** 4.1 ± 1.0 points **3.** 3.9 ± 1.1 points **4.** 3.9 ± 1.2 points Mean ± SD VAS score: **1.** 86.5 ± 15.5 mm

*PROMs* patient-reported outcomes, *SSC* single-suture craniosynostosis, *VAS* Visual Analogue Scale, *AD* average distance, *SP* symmetry percentage, *YQOL-FD* Youth Quality of Life with Facial Differences, *SD* standard deviation, *N/A* not available

### Reported outcome measures of cosmetic satisfaction

Six papers reported on outcome measures that concerned patients’ or their parents’ or caregivers’ personal satisfaction with general, facial or skull appearance using questionnaires or interviews [7, 15, 18, 20–22]. In 2 of these papers, the outcome measures were assessed by the patients only [7, 21], in 3 by the parents or caregivers only [15, 20, 22] and in 1 by both patients and parents or caregivers [18]. Four of the 6 papers used a Visual Analogue Scale (VAS) score to assess cosmetic satisfaction [6, 7, 15, 22], in which a score of 0 mm was considered very unsatisfied and a score of 100 mm corresponded with very satisfied. Salokorpi et al. used this score for patients to answer the question “How satisfied are you with your current facial appearance?”, of which the mean VAS score was 75 mm (range 29 – 100 mm) [7]. Svalina et al. used this score for patients to rate their satisfaction with general and facial appearance, but they did not report the crude VAS scores [21]. Instead, correlation coefficients between VAS scores and facial asymmetry parameters were presented [21]. Two studies used the VAS score to rate the parents’ or caregivers’ satisfaction with skull shape [15, 22]. One of these studies reported a mean score of 5.6 mm (standard deviation [SD] 7.5 mm) and the other study presented a mean score of 86.5 mm (SD 15.5 mm). In 2 of the 6 papers, the patients or their parents or caregivers were asked binary questions to assess cosmetic satisfaction [7, 20]. These included “Is there something that bothers you in your facial appearance?”, “Are you pleased with the cosmetic outcome?” and a rating of whether the patients’ scar bothered them [7, 20]. The answer was “yes” in 32.5%, 86.0% and 10.0%, respectively [7, 20]. Three of the 6 papers used a 5-point scale for assessment of cosmetic satisfaction [15, 18, 22]. Kelleher et al. used this scale for both patients’ and their parents’ or caregivers’ rating of satisfaction with head shape and the bicoronal scar [18]. Head shape was rated as excellent in 29% and very good in 40%, while the bicoronal scar was valued as excellent in 48% and very good in 28% [18]. Two studies used the 5-point scale to help parents or caregivers rate the appearance of skull length, skull width, the forehead and the neck/occiput, in which a higher score corresponded with higher cosmetic satisfaction [15, 22]. The weighted means ± SD’s were 4.13 ± 1.02 for skull length, 4.15 ± 0.98 for skull width, 3.99 ± 1.07 for the forehead and 4.01 ± 1.09 for the neck/occiput.

A total of 2 papers reported on outcome measures that included an aesthetic outcome staging, in which the parents’ or caregivers’ opinion on the cosmetic outcome was evaluated at a minimum of 12 months postoperatively [12, 17]. This opinion was categorized into adequate or non-adequate and added to the treating surgeon’s opinion. Paired with assessment of facial asymmetry and the Whitaker classification, the aesthetic outcome staging was grouped into grade one to four [12, 17]. Grade one and two were characterized by an adequate opinion of the parents or caregivers and corresponded with an excellent or good cosmetic outcome, respectively [12, 17]. The first study reported 94.4% in grade one and 5.6% in grade two [12], while the second study reported 80% in grade one and 20% in grade two [17].

One paper reported on outcome measures that concerned parents’ or caregivers’ evaluation of the postoperative result with special attention to anatomical proportions [19]. A perfect postoperative result with anatomically normal proportions was achieved in 66%, while minor outline irregularities were reported in 23%.

### Reported outcome measures of other patient-reported outcomes

Two papers reported on PROMs that concerned patients’ or their parents’ or caregivers’ opinion on the normalcy and noticeability of their head shape, whether their head shape bothered them and whether other people made comments about it [13, 16]. Dalton et al. used a 10-point scale for parents to answer the questions “How noticeable is your child’s head shape to other people?”, “How much does your child’s head shape bother you?” and a rating of whether surgery made a difference to their child’s head shape [13]. They reported a 3.7 points decrease in noticeability of head shape and a 4.3 points decrease in how much their child’s head shape bothered them pre- versus postoperatively. The mean score of whether surgery made a difference to their child’s head shape was 9.5 points, where a score of 10 corresponded with surgery making it much better. Patients were asked “How noticeable is your head shape to other people?” and “How much does it bother you?”. A mean difference of 1.22 points between younger and older children was reported for noticeability of head shape, while this mean difference was 1.12 points for whether this bothered them [13]. Joly et al. used binary questions for patients that included “Do you find your skull normal?” and “Do other children make comments about it at school?” and they asked parents whether they felt that their child’s skull was normal. The answer was “yes” in 64.7%, 35.3% and 35.3%, respectively [16].

Salokorpi et al. studied PROMs that concerned patients’ general health, educational attainment and employment-, relationship- and housing status following surgical treatment for SSC [7]. They reported that 22.5% patients suffered from migraine, 27.5% suffered from mental health issues, 22.5% lacked professional education, 12.5% were unemployed, 52.5% were in a long-term relationship and 42.5% lived in their own house [7].

Gabrick et al. used the Youth Quality of Life with Facial Differences (YQOL-FD) questionnaire to assess PROMs regarding patients’ quality of life [14]. In this questionnaire, patients score different quality of life domains, including positive and negative consequences of suffering from SSC, coping, negative self-image and stigma [14]. For positive consequences and coping, a higher score corresponded with a superior result, while for negative consequences, negative self-image and stigma, a higher score was associated with a worse result. The mean scores ± SD’s were 38.4 ± 27.8 for positive consequences, 26.6 ± 33.9 for coping, 16.1 ± 21.6 for negative consequences, 8.1 ± 14.1 for negative self-image and 8.5 ± 20.4 for stigma [14].

## Discussion

The current systematic review has provided an inclusive and up-to-date overview of different PROMs following surgical treatment of SSC, including cosmetic satisfaction, self-esteem, social behavior and quality of life.

The majority of selected papers reported on cosmetic satisfaction [7, 12, 15, 17–22]. These studies presented various types of measures for evaluating this outcome, including 1) the use of the VAS score, binary questions or a 5-point scale to rate personal satisfaction with general, facial or skull appearance; 2) the use of an aesthetic outcome staging in which personal opinion was added to the treating surgeon’s opinion; and 3) the use of an evaluation of anatomical proportions of the skull and face. Several studies designed their own questionnaire consisting of general questions regarding cosmetic satisfaction [7, 18, 21]. Other studies assessed patients’ or their families’ satisfaction with aesthetic appearance in more detail, for example by evaluating appearance of skull length and width [15, 19, 22]. The findings of the selected studies showed a trend towards an overall improvement in aesthetic appearance following surgical treatment of SSC, although assessed using different outcome measures [7, 12, 15, 17–20, 22]. One study reported a very low VAS score for satisfaction with skull shape (VAS 5.6 ± 7.5 mm) but concluded that cosmetic satisfaction was good, suggesting the low VAS score to be an inaccuracy in their results [22]. The variability in outcome measures prohibits a meaningful comparison or meta-analysis of the association between cosmetic satisfaction and type of SSC or surgical treatment. Moreover, this variety highlights the need for a uniform approach to evaluate patients’ cosmetic satisfaction. For this, an inclusive and uniform questionnaire to assess patients’ subjective cosmetic satisfaction would be beneficial. Ideally, such a questionnaire would incorporate detailed data on both patients’ own cosmetic satisfaction at different stages of life (i.e., childhood, adolescence and adulthood) as well as their families’ opinion. These results are useful for patients’ families and physicians to include in preoperative counseling on the optimal management of SSC [11, 23].

Besides a need to evaluate patients’ perceived cosmetic satisfaction, it is also essential to consider the importance of their aesthetic appearance and how this may influence their social behavior and quality of life [24]. A retained cosmetic deformity might draw negative attention from strangers or peers and subsequently lead to self-consciousness and social withdrawal [7, 13, 24]. Four papers reported on PROMs other than cosmetic satisfaction [7, 13, 14, 16]. A wide range of different PROMs were described, including patients’ long-term postoperative general health and socioeconomic status [7], patients’ and their families’ rating of the normalcy and noticeability of their skull and facial appearance and how much this bothered them [7, 13, 16], and patients’ answers to the YQOL-FD, a validated questionnaire to assess quality of life [14]. Based on the broad range of reported PROMs, no clear overall trend of the results could be identified. Favorable PROMs results were reported for general health and socioeconomic status. Although a relatively high number of patients reported on headaches or mental health problems following surgical treatment of SSC, this number did not differ significantly from sex- and age-matched controls [7]. Furthermore, a significant decrease in the noticeability of head shape and the extent to which this bothered patients and their families was found [13]. Interestingly, one study found that patients report their head shape as significantly more noticeable than their parents [13], while a different paper found that patients more often feel like their skull is normal than their parents do [16]. In addition, older patients reported their head shape to be more noticeable and were more bothered than younger patients, reflecting a possible increased awareness of aesthetic appearance during puberty and adolescence [13, 25]. These results emphasize the need to evaluate both patients and their families at different stages of life to help offer psychosocial support resources to those most in need. Less favorable PROMs results were identified using the YQOL-FD [14]. These findings showed that patients who underwent surgical treatment of SSC lacked adequate coping strategies for dealing with a visible facial difference during adolescence [14]. Furthermore, fairly low scores were measured for the domain of positive consequences. Since both domains of coping and positive consequences are often related to social interactions and relationships, these results suggest that undergoing surgical treatment of SSC may considerably influence patients’ sense of belonging and social experience [14]. Nevertheless, favorable scores for the domains that encompass the individual sense of self and internalized emotions were reported, suggesting that patients are comfortable with themselves [14]. The variability in reported PROMs impedes a useful comparison or meta-analysis of the association between PROMs and type of SSC or surgical treatment. Just as for evaluating cosmetic satisfaction, a comprehensive and uniform questionnaire to assess different PROMs would be valuable. Dalton et al. introduced a novel questionnaire for collecting uniform data of patients’ and their families’ perception of head shape and demonstrated a remarkably high return rate [13]. However, this method has not been implemented elsewhere yet. Moreover, an inclusive questionnaire on PROMs would ideally include additional data on general health status, socioeconomic status, level of self-esteem and sense of belonging, social behavior and experience, and overall health-related quality of life.

### Strengths and limitations

An important strength of this study is that it has provided an inclusive, up-to-date and in-depth summary of the existing literature related to cosmetic satisfaction and other PROMs of patients who underwent surgical treatment of SSC. This literature review has highlighted opportunities for further research to expand and improve methods to assess patients’ and their families’ cosmetic satisfaction and other PROMs. Nevertheless, this study has some limitations. First, only papers written in English and of which the full text was available were included, which could have caused publication bias. Second, substantial heterogeneity was found among the included papers regarding the outcome measures used to assess cosmetic satisfaction and other PROMs, thus prohibiting a useful meta-analysis from being conducted.

## Conclusion

Following surgical treatment of SSC, there is a trend towards an overall improvement in aesthetic appearance. Beyond this, our systematic review illuminates that the existing literature contains a wide variation in outcome measures for evaluating cosmetic satisfaction and other PROMs of patients who underwent surgical treatment of SSC. Further research is warranted on developing an inclusive and uniform approach to assess patients’ subjective cosmetic satisfaction and other PROMs to help optimize preoperative counseling and psychosocial care for patients and their families.

## Data Availability

Data sharing not applicable to this article as no datasets were generated or analyzed during the current study.
